# Psychometric properties of the English version of the Audio Processor Satisfaction Questionnaire (APSQ)

**DOI:** 10.1371/journal.pone.0273390

**Published:** 2022-09-01

**Authors:** Jenna Felder, Edda Amann, Ilona Anderson, Joshua Stohl, Mareike Billinger-Finke

**Affiliations:** 1 North American Research Laboratory, MED-EL Corporation, Durham, North Carolina, United States of America; 2 MED-EL GmbH, Innsbruck, Austria; Hannover Medical School: Medizinische Hochschule Hannover, GERMANY

## Abstract

**Objective:**

The Audio Processor Satisfaction Questionnaire (APSQ) is a standardized tool to measure a user’s satisfaction with their audio processor(s). It was first developed and validated in the German language. The purpose of the current study was to validate the English version of the APSQ.

**Design:**

The 15 items of the APSQ were translated into English. Item and scale analyses assessed the quality of individual items and of the questionnaire in its entirety.

**Study sample:**

Sixty-seven adults with hearing implants participated. Forty-six of them completed the questionnaire twice within 2–4 weeks.

**Results:**

High mean values were obtained with total scores and with scores of the comfort, social life, and usability domains, indicating that users are generally satisfied with their audio processors. The questionnaire achieved good test-retest reliability with high internal consistency. A significant positive correlation between time since implantation and user satisfaction was found.

**Conclusion:**

Results of the item and reliability analyses suggest that the English version of the APSQ is a valid and reliable tool to assess user satisfaction with their audio processor(s).

## Introduction

Hearing implants (HIs) such as cochlear implants (CIs), middle ear implants, and bone conduction implants have been designed to improve communication abilities and quality of life for individuals who are deaf or hard of hearing. These implants were shown to improve speech understanding [1], music appreciation [2], quality of life [3, 4] and general communication [5].

Manufacturers of HIs not only want to show that individuals can benefit from their devices, they also need to be aware of whether their users are satisfied with their products and how to improve devices to meet their users’ needs. Several questionnaires have been designed to measure various facets of user satisfaction. The Speech, Spatial and Qualities of Hearing Scale (SSQ) [6] and the Hearing Implant Sound Quality Index (HISQUI19) [7] were created to evaluate the subjective quality of hearing for HI users. The Cochlear Implant Function Index (CIFI) [8] was created to assess quality of life in real-world situations (e.g. telephone use, communication at work, hearing in noise, etc.). Beyond sound quality and quality of life, in the hearing aid industry the Satisfaction with Amplification in Daily Life (SADL) [9] was developed to quantify general satisfaction with hearing aids. In the cochlear implant industry, several questionnaires have been developed to measure user satisfaction for specific audio processors, such as the Neptune audio processor [10], the TEMPO+ audio processor [11], and the RONDO single-unit audio processor [12]. Instead of creating a unique questionnaire for each audio processor, it became clear that a single questionnaire was needed that could be used for any audio processor. For this reason, Billinger-Finke et al. (2020) [13] created the Audio Processor Satisfaction Questionnaire (APSQ), a specific and standardized tool to measure a user’s satisfaction with their audio processor. The German language version of the APSQ has been validated with users of different types of HIs (e.g. cochlear implants, middle ear implants, bone conduction devices, etc.) and therefore is applicable to all types of HIs and independent of the model or generation of the audio processor. The APSQ measures user satisfaction with the audio processor for three domains, described as subscales by Billinger-Finke et al. (2020) [13]: comfort, social life, and usability. Items (questions) are intended to evaluate how comfortable the audio processor is to wear, how well it works in various social settings, and whether the audio processor is easy to use. The expert group that created the questionnaire decided that the three-domain structure should be implemented as it could provide a more detailed insight into user satisfaction than does a total score alone. The names of three domains were motivated by the content of their items.

A major task of medical professionals who fit HIs is getting users to feel comfortable with wearing the audio processor in their everyday life and to make sure that the user benefits from the device. Informational counseling is provided to help the HI user acclimate to using new equipment and involves showing the HI user how to turn the audio processor on and off, how to change batteries, how to put the processor on and taking it off, and making sure that the device fits comfortably on the head and/or ear. However, it is well-documented that patients retain little information from clinical appointments [14, 15]. The APSQ is a tool that can be provided to HI users prior to their appointment and can guide medical professionals regarding what information or topics should be addressed or re-discussed during an appointment. For HI users, the APSQ can give them a voice to share information with the medical professional fitting their device.

The APSQ was first developed and validated in the German language (German title: Audioprozessor-Zufriedenheitsfragebogen) [13]. The German language version of the APSQ was directly translated into English by a professional translator. This version was proofread by two reviewers (one native German speaker with proficiency in English and one native English speaker with proficiency in German). The final version of the English language version was then checked by another native English speaker. The final English language translation of the APSQ was published in the original manuscript [13]. That translation was used in the current project. The purpose of the current project was to validate the English language version of the APSQ. There were two goals for the current study: (1) Obtain psychometric measures for the English version of the APSQ and (2) compare the psychometric measures of the English language version with those obtained from the German language version.

## Methods

### Participants

A total of 67 participants were included in this study (33 male, 34 female), with a mean age of 64 years (standard deviation = ±11.92 years; range: 20–88 years) when they completed the questionnaire for the first time. Criteria for inclusion were: (1) age of 18 years or older; (2) implanted with a MED-EL hearing system (MED-EL GmbH, Innsbruck, Austria); and (3) native speaker of English. Participants were recruited through the North American Research Laboratory (MED-EL Corp., Durham, North Carolina, United States) and through the North American HearPeers community. This research study was approved by the Western Institutional Review Board (WIRB; Protocol #20100066). Signed and dated informed consent forms were obtained from each participant prior to their participation in the research study. No adverse events were reported during the course of the study.

In total, there were 94 HIs for all 67 participants. As can be seen in Table 1, of the 67 participants, 27 (40.3%) were bilateral CI users (54 ears). The remaining 40 (59.7%) participants were unilateral users, and all but 2 were CI users. One of the two users was implanted with the MED-EL BoneBridge hearing system in the left ear, the other used ADHEAR in the right ear. Of the 38 unilateral CI users, 20 were bimodal with a hearing aid in the contralateral ear, 9 reported single-sided deafness with normal hearing in the contralateral ear, and 9 reported hearing loss but no device in the contralateral ear.

**Table 1:** Demographic information comparing the current English dataset to the German dataset (Billinger-Finke et al., 2020), including the hearing systems and audio processors used, along with the device usage times, per ear. EAS = electric acoustic stimulation.

	English data		German data (Billinger-Finke et al., 2020)	
	Left ear	Right ear	Left ear	Right ear
Normal hearing	3	5	4	2
Hearing loss	64	62	64	66
**HEARING SYSTEM**:				
Hearing aid	6	4	16	10
Cochlear implant	46	43	36	41
EAS system	1	2	4	5
Middle ear implant	1	-	6	6
Bone conduction system	-	1	1	1
No hearing aid system	13	17	5	5
**AUDIO PROCESSOR**:				
SONNET	30 (46.9%)	29 (46.8%)	21 (32.8%)	25 (37.9%)
SONNET 2	5	3	-	-
SONNET EAS	1	2	-	-
OPUS 2	-	2	16	15
RONDO	7	5	1	4
RONDO 2	4	4	-	-
SAMBA	1	-	5	3
ADHEAR	-	1	-	-
DUET 2	-	-	1	-
AP 404	-	-	-	1
Amadé	-	-	2	3
No audio processor	7	13	21	15
**DEVICE USAGE TIME**:				
0–3 h	-	-	2	0
3–6 h	1	-	-	-
6–9 h	1	-	3	3
9–12 h	9	8	6	10
>12 h	37	37	42	41
Missing	19	22	15	14

On average, participants had their hearing system for 4.7 years (SD ±3.41 years; range: 3 months-16 years). Device usage time was reported for 93 ears, and of those responses, 79% indicated more than 12 hours of audio processor use per day. One participant did not report device usage time for their implanted ear. The 40 unilateral participants reported either no device use or hearing aid use in one ear and thus did not fill out device usage time for the non-implanted ear. Further information on the hearing systems and audio processors used, along with device usage times, are shown in Table 1.

### The questionnaire

The preliminary version of the APSQ used a 5-point Likert scale and started with 21 items in German; however, based on the preliminary German-language validation, the final questionnaire only included 15 items and was switched to a visual analogue scale (VAS) [16], ranging from 0 to 10 to get a continuous measure of device satisfaction (see Billinger-Finke et al. 2020 for more information on the development of the questionnaire) [13].

The English language version of the APSQ that was evaluated in the current study was the translation of the final 15-item German language questionnaire as published in Billinger-Finke et al. (2020) [13]. Answers to the 15 items are given on a VAS ranging from 0 to 10. A value of 0 corresponded to a response of “does not agree at all” and 10 as “fully agrees.” If an item did not apply to the participant, they were able to check a “not applicable” checkbox instead of using the VAS. The items were classified into three domains in terms of content and each domain contained 5 items: (i) comfort (items 3, 6, 9, 12, and 15), (ii) social life (items 1, 4, 7, 10, and 13), and (iii) usability (items 2, 5, 8, 11, and 14). The domain score was obtained by calculating the average score of the 5 items. The total score was obtained by calculating the average score of all items.

During the study, participants received two electronic copies of the APSQ written in English. The first copy of the questionnaire was to be completed immediately after signing the informed consent form. Approximately 2–4 weeks after completing the first questionnaire, each participant was asked to fill out the questionnaire for a second time to allow for the analysis of test-retest reliability. Questionnaires were only included in the reliability analyses if the second questionnaire was completed within the defined time frame of 2–4 weeks. Both copies of the APSQ were sent and returned electronically via email.

Participants were asked to fill out the questionnaire to the best of their ability. The participants with a unilateral audio processor answered items for the non-implanted ear as ‘not applicable’. Participants with bilateral audio processors answered the 15 items twice, once per ear, allowing for any differences between the two ears to be captured. This differed from Billinger-Finke et al. (2020) [13] where the bilateral participants answered the 15 items once rather than once per ear. In order to make comparisons between the two datasets, the scores for each ear obtained with the English questionnaire were averaged for each item and the averaged score was subsequently used for statistical analysis.

Of the 15 items, 9 had missing data with between 1 and 5 instances in which the items were either not answered or answered as ‘not applicable.’ Missing data were treated as ‘missing values.’ Consistent with Billinger-Finke et al. (2020) [13], questionnaires were excluded if three items or more were not answered. In the current study, no questionnaire had more than three items that were not answered or answered as ‘not applicable’, and thus no participants were excluded from the validation analyses. Of the 67 participants, 52 had zero items missing, 7 had one item missing, 6 had two items missing, and 2 had three items missing.

### Statistical analysis methods

The psychometric characteristics of the items of the APSQ were evaluated with the classical test theory model [17, 18]. Additional analyses were completed to investigate the impact of different variables on user satisfaction as depicted by the total score, calculated by averaging the VAS rating for all 15 items. Time since implantation, age, type of audio processor, type of hearing system and device usage time were correlated with the APSQ total score applying Pearson correlation or Spearman`s rho. The influence of gender was examined using the Mann-Whitney U-test. To check the distribution of the data, the Kolmogorov-Smirnov test and the Shapiro-Wilk test were used. A p-value of 0.05 or less was considered statistically significant. Statistical analyses were completed using IBM SPSS Statistics v24 (IBM, Armonk, New York. US).

## Results

The participants used a broad range of the VAS to answer the items in the questionnaire. The mean scores were 8.1 (SD ±1.61) for the ‘comfort’ domain, 8.3 (SD ±1.95) for the ‘social life’ domain, and 8.9 (SD ±1.26) for the ‘usability’ domain. Overall, the mean scores per item ranged between 6.2 and 9.5 (SD between ±1.00 and ±3.16) with a tendency towards a ceiling effect.

### Item analysis

Item discrimination and item homogeneity were examined in order to assess the quality of the items and of the questionnaire as a whole.

#### Discrimination index

The discrimination index measures the validity of an item, how the item correlates with the total score of all items, and the item’s ability to discriminate between high scorers and low scorers. If an item has a high correlation with the total score (i.e. the average of all items), then it is able to differentiate between those participants who are “more satisfied” giving a high rating on the VAS and those participants that may be “less satisfied” and give a lower rating for a particular item. The item discrimination classification is based on the results of the corrected item-total correlation. This is the correlation (Pearson correlation) between each item and the total score that excludes that item. According to Ebel and Frisbie (1991) [19], a correlation of 0.40 or higher corresponds to very good items, 0.30 to 0.39 are reasonably good but could possibly be improved, 0.20 to 0.29 are marginal items and need to be revised, and items below 0.19 are considered poor and need to be majorly revised or removed [19]. As shown in Table 2, results indicated that the items were generally rated as ‘very good’ in terms of item discrimination. Item 15 was rated as ‘reasonably good’ with a correlation of 0.39. When comparing the item-total correlations between the English and the German datasets in Table 2, it was noted that within the “social life” domain, the correlations were higher for all 5 items in the English dataset compared to the German dataset.

**Table 2:** Results from the item discrimination test. AP = audio processor.

			English data		German data (Billinger-Finke et al., 2020)	
Domain	Item no.	Item labels	Corrected Item–Total correlation	Discrimination	Corrected Item–Total correlation	Discrimination
Comfort	3	AP is skin friendly	0.679	Very good	0.254	Marginal
	6	AP is comfortable to wear	0.674	Very good	0.669	Very good
	9	Can wear glasses comfortably with AP	0.537	Very good	0.431	Very good
	12	Can wear hats and helmets comfortably with AP	0.500	Very good	0.691	Very good
	15	AP stays in the same position all day	0.390	Reasonably good	0.639	Very good
Social Life	1	Feel safer when wearing AP	0.752	Very good	0.337	Reasonably good
	4	AP allows for a physically active lifestyle	0.673	Very good	0.527	Very good
	7	AP helps live a more independent life	0.696	Very good	0.529	Very good
	10	AP makes cultural activities more enjoyable	0.592	Very good	0.523	Very good
	13	AP makes social activities more enjoyable	0.662	Very good	0.393	Reasonably good
Usability	2	Easy to put AP in correct place on head	0.749	Very good	0.710	Very good
	5	Easy to change the batteries of AP	0.472	Very good	0.514	Very good
	8	Easy to switch AP on and off	0.513	Very good	0.263	Marginal
	11	AP functions properly	0.655	Very good	0.655	Very good
	14	Easy to clean and dry AP	0.580	Very good	0.517	Very good

#### Item homogeneity

Item homogeneity evaluates the extent to which the individual items correlate with the total score. A high correlation is obtained when more single items correlate with the total score and with lower variability across these correlations [20]. Items are considered homogenous when correlations are significant, reflecting that the questionnaire items are measuring the same underlying construct, such as user satisfaction. This analysis revealed that all items correlated significantly with the total score (Pearson correlation: *p* < 0.001), showing good item homogeneity. These results are consistent with the findings of the German language version, in which all items correlated significantly with the total score (Pearson correlation: p<0.001 to p = 0.005).

### Scale analysis

#### Reliability

Cronbach’s alpha [21] and the Guttman split-half-coefficient were used to test the questionnaire’s internal consistency. Guttman’s split-half-coefficient estimates the full test reliability of the questionnaire. It is calculated by splitting the dataset into two halves and correlating them. A coefficient of 0.7 or above is considered an acceptable level for internal consistency. The English version of the APSQ reached a good reliability with high internal consistency (Cronbach’s α = 0.90; Guttman’s split-half coefficient = 0.90), consistent with the findings of the German version (Cronbach’s α = 0.84; Guttman’s split-half coefficient = 0.86) [13].

Kendall`s Tau-b coefficient was used to measure reliability as an indication of the consistency and repeatability of the questionnaire across time. This measure shows how likely it is that a participant would obtain the same score when they complete the test again. According to Kelley (1939) [22], a coefficient of 0.50 or below has questionable reliability, whereas a coefficient greater than 0.80 is very good. For the test-retest reliability analyses, the data collected from 46 participants who completed the questionnaire twice were analyzed. Five participants were excluded from the reliability analysis as they completed the second questionnaire outside of the defined time frame of 2–4 weeks. Sixteen participants did not fill out and/or return the second questionnaire. The results of the test-retest reliability analysis were significant (Kendall`s Tau-b: *r* = 0.724; *p*<0.001), confirming repeatability and consistency of the questionnaire across time. These results are consistent with the findings of the German language version (Kendall`s Tau-b: *r* = 0.817; *p*<0.001) [13].

#### Construct validity

To test the suitability of the items for factor analysis, the KMO test [23] and the Bartlett test of sphericity were performed as measures of sampling adequacy. The KMO test reached a value of 0.79, which is considered ‘acceptable’ according to Kaiser et al. (1974) [23]. The result of the Bartlett test of sphericity indicated a significant correlation between the items (χ^2^ = 519.669, df = 105, *p* < 0.001). The results of both tests indicate suitability of the data for factor analysis.

Factor analysis with a rotated quartimax factor solution (extraction method: principal component analysis) was used to check the underlying factor structure of the items [24]. Factor loadings with absolute values greater than 0.40 were considered significant and assigned to the appropriate factor, and those below 0.40 were suppressed. As can be seen in Table 3, the items loaded onto three components (i.e., factors) which explained 66.8% of the total variance (Table 4). The five items of the domain ‘social life’ loaded on the first component, and the five items of the domain ‘usability’ loaded on the second component. Three items of the domain ‘comfort’ loaded on the third component, and items 3 and 12 loaded on components 2 and 1, respectively (see Table 3). Factor loadings reflect that the items of the domain ‘social life’ depict a similar construct and the items of the domain ‘usability’ depict a second construct. The items of the domain ‘comfort’ show greater variation.

**Table 3:** Results from the factor analysis with a rotated quartimax factor solution (extraction method: Principal component analysis). Other factor loadings are marked in grey. C = components (i.e. factors); AP = audio processor.

			Factor loadings English data			Factor loadings German data (Billinger-Finke et al., 2020)		
Domain	Item no.	Item labels	C 1	C 2	C 3	C 1	C 2	C 3
Comfort	3	AP is skin friendly	.428	0.601		0.551		
	6	AP is comfortable to wear		.472	0.715	0.871		
	9	Can wear glasses comfortably with AP			0.786	0.598		
	12	Can wear hats and helmets comfortably with AP	0.724			0.574		
	15	AP stays in the same position all day			0.657	0.852		
Social Life	1	Feel safer when wearing AP	0.915				0.727	
	4	AP allows for a physically active lifestyle	0.694			0.557		
	7	AP helps live a more independent life	0.840				0.719	
	10	AP makes cultural activities more enjoyable	0.615				0.810	
	13	AP makes social activities more enjoyable	0.890				0.843	
Usability	2	Easy to put AP in correct place on head	.409	0.594	.490	0.747		
	5	Easy to change the batteries of AP		0.868		0.711		
	8	Easy to switch AP on and off		0.656				0.798
	11	AP functions properly	.487	0.585		0.820		
	14	Easy to clean and dry AP		0.733		0.632		

**Table 4:** The total variance explained by each component.

Component	Initial Eigenvalues		
	Total	% of Variance	Cumulative %
1	6.922	46.147	46.147
2	1.808	12.057	58.204
3	1.285	8.566	66.770
Component	Rotation Sums of Squared Loadings		
	Total	% of Variance	Cumulative %
1	4.714	31.429	31.429
2	3.395	22.636	54.065
3	1.906	12.705	66.770

### Relationship between additional factors and the total score

**Fig 1:**
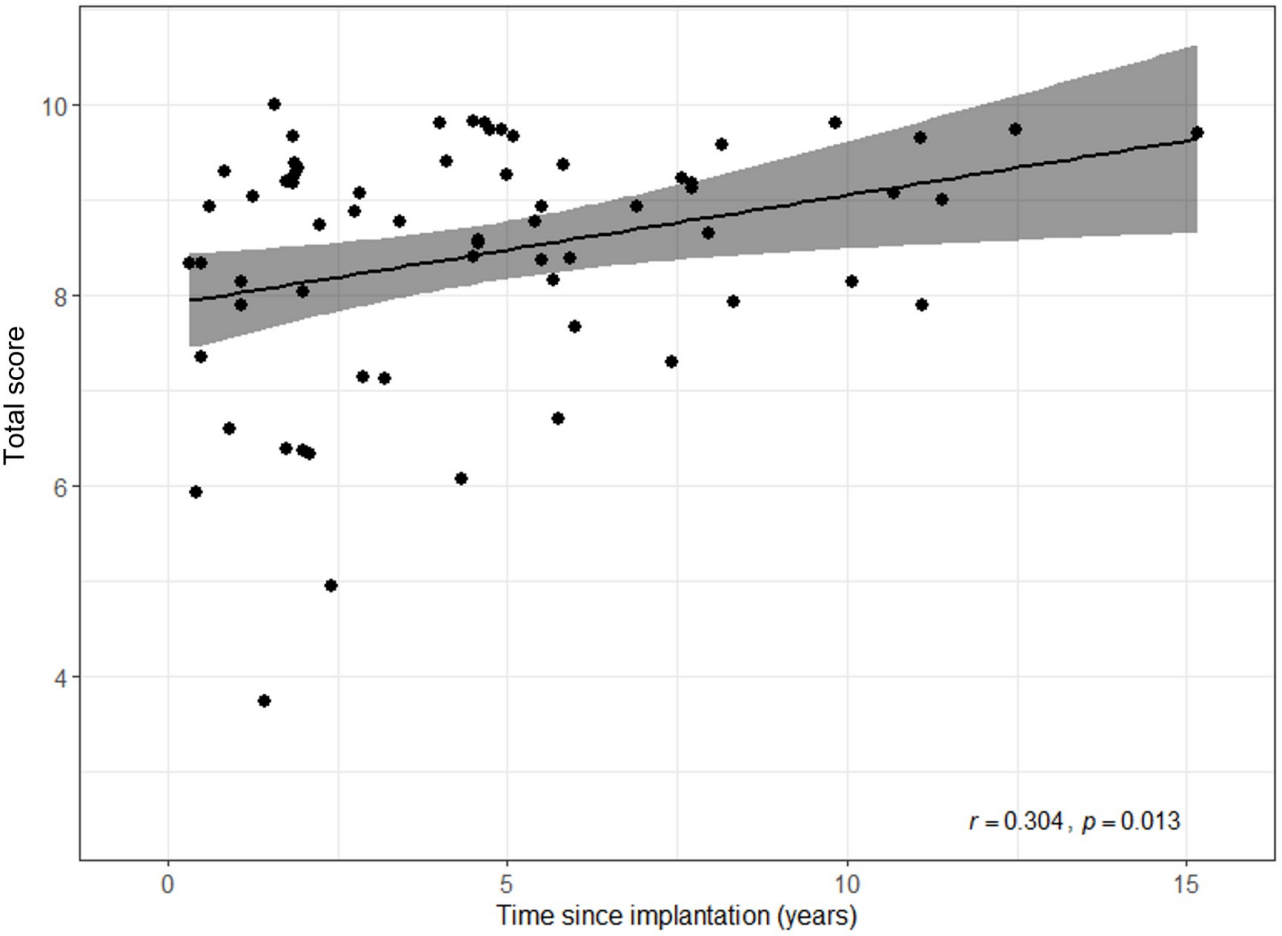
The correlation between time since implantation and the total score.

Hypotheses were not formulated a-priori regarding the relationship between additional factors and the total score, as the primary aim of this study was to evaluate the psychometric properties of the English language version of the APSQ and compare those results with the German language version of the APSQ. As an additional point of interest, we examined a possible relationship between different variables and user satisfaction (as depicted by the total score). As can be seen in Fig 1, time since implantation and user satisfaction correlated significantly (Pearson *r* = 0.304; *p* = 0.013). No significant correlation was found between the participants’ age and user satisfaction (*r* = 0.030; *p* = 0.808). Type of audio processor (Spearman rho: *r* = 0.196, *p* = 0.111), type of hearing system (Spearman rho: *r* = 0.123, *p* = 0.323), and device usage time (Spearman rho: *r* = 0.186, *p* = 0.135) did not correlate significantly with user satisfaction. Gender did not have a significant effect on user satisfaction (Mann-Whitney U-test: *u* = 509.50; *p* = 0.518). In summary, time since implantation was the only factor that showed a significant relationship with user satisfaction.

## Discussion

The purpose of the current project was to obtain psychometric measures for the English language version of the APSQ. Many studies have been conducted using non-validated questionnaires attempting to measure user satisfaction with an audio processor [10–12]. Motivated by the need for a validated questionnaire that could be used for all types of HIs and audio processors, Billinger-Finke et al. (2020) [13] designed the APSQ. The German language version of the APSQ provides manufacturers, clinicians and researchers with a validated, user-friendly questionnaire that can be used to quantify HI users’ self-perceived satisfaction with their audio processor(s) in their everyday life. The current study aimed to validate the English version of the APSQ by collecting data from English-speaking HI users.

Participants with bilateral audio processors answered the items for each ear and the scores for each item were averaged. This method of averaging allowed for the comparison to the German version of the APSQ, where the bilateral participants answered the 15 items once rather than once per ear. For all participants, the difference in score on a given item was never greater than two points between the two ears. However, it is plausible that answers to some questions, particularly the questions within the usability domain, are more likely to differ between the two ears. Question 2, “It is easy to put the audio processor back on its proper place on my head” and Question 8, “It is easy to switch the processor ON and OFF,” for example, may depend on whether the user is right-handed or left-handed. More data would need to be obtained on bilateral HI users to determine whether responses differ between the two ears.

The 5-point Likert scale used in the preliminary German questionnaire version was transformed into a visual analogue scale (VAS range 0–10) to get a continuous measure of device satisfaction [16]. From the participant’s perspective, the VAS appears continuous and allows for a broader range for rating device satisfaction. If the data obtained using a VAS scale shows normal distribution, the VAS is generally accepted as an interval or ratio scale, so that parametric statistics can be applied. The Kolmogorov-Smirnov test and the Shapiro-Wilk test revealed normal distributions of the data for both the German and English language versions of the APSQ, suggesting the VAS scale is an appropriate scale to use for this questionnaire.

The item analysis consisted of item discrimination and item homogeneity measures. Of the 15 items, 14 had very good item discrimination [19] and as for item homogeneity, all items were statistically significant. Although the word “satisfaction” is never used in any of the APSQ items, the construct validation supports the assertion that the total score quantifies user satisfaction with their audio processor(s). Questionnaire reliability was measured through internal consistency and by comparing test-retest reliability. According to Cronbach’s alpha and the Guttman split-half coefficient, the questionnaire achieved good reliability with high internal consistency. The results of the test-retest reliability analyses were significant, confirming repeatability and consistency of the users’ responses when filling out the questionnaire twice within a 2–4 week timeframe. These results confirm that the English version of the APSQ is a valid and reliable tool.

The second goal of the current study was to compare the psychometric measures of the English language version with those obtained from the German language version of the APSQ. The findings of the item analysis are consistent with the findings in the German dataset [13]; however, correlations for the item discrimination within the “social life” domain were generally higher in the English dataset. This suggests that the English items provided better differentiation between users who were ‘satisfied’ (i.e. higher scores) and those who were ‘less satisfied’ (i.e. lower scores) within the “social life” domain. In regard to reliability, both the English and German language versions of the APSQ reached a good reliability and high internal consistency.

High values were obtained in total scores and within each of the three domains, with mean scores of 8.1, 8.3, and 8.9 for the comfort, social life, and usability domains, respectively. These findings are consistent with findings obtained with other questionnaires in the literature, finding that HI users are generally satisfied with their audio processors [11, 12]. While the total score on the APSQ is a measure of overall satisfaction, the APSQ also offers the ability to separate scores into three domains (comfort, social life, and usability). Within the “social life” domain, comparisons could possibly be made to other validated tools such as the SSQ [6], HISQUI19 [7], CIFI [8], and the Hearing Handicap Inventory for Adults (HHIA) [25] designed to measure “quality of hearing” and “quality of life.” What differentiates the APSQ questions within the “social life” domain from these other tools is that the questionnaire asks how the audio processor itself contributes to these specific social situations (e.g. “My processor makes it easier for me to enjoy social activities”). Answers to these questions likely vary based on the specific audio processor and features that are available. Features such as microphone directionality, noise reduction, connectivity, and the audio processor type (e.g. single-unit processor versus behind-the-ear) vary and may result in different levels of user satisfaction within the “social life” domain of the APSQ.

The “usability” domain of the APSQ was designed to evaluate the ease of use as a component of user satisfaction with the audio processor. If the device is easy to use, the result should lead to increased user satisfaction. Feedback within this domain, as well as in the “comfort” domain can give the manufacturer hints for specific improvements that can be made to their products. While we are unaware of other tools that assess various aspects of “comfort” with wearing a HI audio processor, the domain of “usability” translates well beyond the use of hearing devices. The classic System Usability Scale (SUS) designed by Brooke (1996) [26] was intended to measure the usability of any tool or system. Although the questions of the SUS are not specific to audio processors as with the APSQ, it is likely that responses on the SUS and within the “usability” domain of the APSQ would be similar, but this would need to be tested to confirm. Questions relating to usability are of increasing importance to medical device manufacturers following medical device regulations requiring they evaluate the usability of their medical devices as it relates to safety (IEC 62366) [27]. The questions within the “usability” domain of the APSQ were not designed with this intention but could potentially be useful within the scope of regulations for medical devices, such as clinical trials or post marketing clinical follow up studies. A follow up study would be needed to see how the “usability” domain within the APSQ compares to other tools used for measuring “usability” within the medical device industry.

For the factor analysis of the English data, all items of the “social life” domain loaded onto the first component (i.e., factor), suggesting that these items all belong to the same construct and that this construct accounts for the highest proportion of the variance in the data. All items of the “usability” domain loaded onto the second component, and the “comfort” domain showed greater variation, with three items loaded onto the third component. Two items (3 &12) in the “comfort” domain loaded onto separate components compared to the three other items in this domain. Those two items were determined to be ‘very good’ from the item analysis, and as the total score is of main interest, those two items need not be re-allocated.

When comparing the factor analysis of the German and English language versions of the APSQ, all items loaded onto three components for both the English and German APSQ, reflecting the total score depicting device satisfaction according to the selected domains in both datasets. In the German dataset, all items from the “comfort” domain, and four out of five items in the “usability” domain loaded onto the first component, suggesting the same underlying construct for these two domains and a higher relative importance compared to the “social life” domain, in which four of five items loaded onto the second component. Although differences in the factor loadings between the German and English datasets are noted, a more systematic study would be required to better control the two samples in order to draw conclusions about their meaning. One of the primary differences between the two samples is that Billinger-Finke (2020) [13] included a larger variety of non-CI users than the current dataset, and although the inclusion criteria were identical to that of Billinger-Finke (2020) [13] with the exception of native language, unfortunately few non-CI users participated in the current study. While it was intended to show that the English language version of the APSQ is applicable to all types of HIs, it is impossible to make this conclusion based on the current dataset. Until more data is obtained with other HIs and from other manufacturers, the English language version of the APSQ should be considered a tool for assessing user satisfaction with MED-EL CI audio processors.

The factor analysis only partially justified the pre-selected domains. In the English version, all items in the ‘social life’ and ‘usability’ domains loaded exclusively onto the first two constructs, respectively, supporting the grouping of these items under common labels. On the other hand, loadings in the ‘comfort’ domain varied, and the variance explained by the second and third constructs is relatively low. Support for the three domains is weaker in the German dataset, with only a single item loading on the third construct. Items from the ‘comfort’ and ‘social life’ domains generally loaded onto separate constructs, with items from the ‘usability’ domain primarily loading onto the first construct with items in the ‘comfort’ domain. More data are required to know if the inclusion of non-CI devices in the German dataset is relevant to the factor analysis, and the total score is evidently more reliable than domain-specific scores for both language versions of the questionnaire. In spite of the relatively weak support for the pre-selected domains, health care professionals, researchers, and manufacturers may still find the domains useful for counseling patients, characterizing satisfaction with new technologies, and improving devices.

Correlation analyses were completed to investigate the relationship of different variables (i.e. age, gender, type of audio processor, time since implantation, device usage time) to user satisfaction. No significant correlations were found between the variables and the total score, except for time since implantation. The significant correlation with time since implantation may be related to increased experience with an audio processor. As users gain more experience wearing and utilizing the audio processor, some of the items describe behaviors that may start to become routine, resulting in higher satisfaction scores over time. It is also possible that the correlation between satisfaction and time since implantation reflects increased user satisfaction due to audio processor improvements and upgrades over time. That is, some participants could have worn an audio processor for many years and recently upgraded to a new device. In this case, overall satisfaction could be related to the new device rather than overall HI experience. We are unable determine if the result in either dataset is related to experience, access to new technologies, or both. Future studies could further investigate this association between time since implantation and user satisfaction by collecting data regarding audio processor use longitudinally, such as when participants upgrade to new technology and how long they’ve used their current audio processor.

## Conclusion

The English language version of the APSQ is a valid and reliable tool to assess user satisfaction with their audio processor(s). With the APSQ now validated in both German and English, it is more accessible to manufacturers, clinicians, and researchers worldwide.

## Supporting information

S1 File The English language version of the Audio Processor Satisfaction Questionnaire (APSQ). (PDF)

S2 File The raw data obtained in the current study. (XLSX)
